# Programmed Intermittent Epidural Bolus versus Continuous Epidural Infusion in Major Upper Abdominal Surgery: A Retrospective Comparative Study

**DOI:** 10.3390/jcm10225382

**Published:** 2021-11-18

**Authors:** Yeon-Ju Kim, Do-Kyeong Lee, Hyun-Jung Kwon, Hye-Mee Kwon, Jong-Hyuk Lee, Doo-Hwan Kim, Sung-Moon Jeong

**Affiliations:** Department of Anesthesiology and Pain Medicine, Asan Medical Center, University of Ulsan College of Medicine, 88 Olympic-ro 43-gil, Songpa-gu, Seoul 05505, Korea; yjk88@amc.seoul.kr (Y.-J.K.); 135060@naver.com (D.-K.L.); hyunjung.kwon1207@gmail.com (H.-J.K.); hyemee.kwon@amc.seoul.kr (H.-M.K.); leejhpain@amc.seoul.kr (J.-H.L.); anesjsm@amc.seoul.kr (S.-M.J.)

**Keywords:** programmed intermittent epidural bolus, continuous epidural infusion, upper abdominal surgery, epidural anesthesia, postoperative analgesia

## Abstract

Although recent evidence shows that the programmed intermittent epidural bolus can provide improved analgesia compared to continuous epidural infusion during labor, its usefulness in major upper abdominal surgery remains unclear. We evaluated the effect of programmed intermittent epidural bolus versus continuous epidural infusion on the consumption of postoperative rescue opioids, pain intensity, and consumption of local anesthetic by retrospective analysis of data of patients who underwent major upper abdominal surgery under ultrasound-assisted thoracic epidural analgesia between July 2018 and October 2020. The primary outcome was total opioid consumption up to 72 h after surgery. The data of postoperative pain scores, epidural local anesthetic consumption, and adverse events from 193 patients were analyzed (continuous epidural infusion: *n* = 124, programmed intermittent epidural bolus: *n* = 69). There was no significant difference in the rescue opioid consumption in the 72 h postoperative period between the groups (33.3 mg [20.0–43.3] vs. 28.3 mg [18.3–43.3], *p* = 0.375). There were also no significant differences in the pain scores, epidural local anesthetic consumption, and incidence of adverse events. Our findings suggest that the quality of postoperative analgesia and safety following major upper abdominal surgery were comparable between the groups. However, the use of programmed intermittent epidural bolus requires further evaluation.

## 1. Introduction

Epidural analgesia is associated with a significant improvement in postoperative pain control, lower requirement of opioids, and enhanced clinical outcomes compared with parenteral opioids after major abdominal surgery [1,2]. Continuous epidural infusion (CEI) and patient-controlled epidural analgesia (PCEA) bolus are generally the traditional methods for epidural analgesia. However, CEI has some disadvantages, a limited area of distribution of the local anesthetic, resulting in a limited extent of the analgesic area and increased consumption of local anesthetic [3]. An alternative infusion strategy that delivers local anesthetic and opioid as a programmed intermittent epidural bolus (PIEB) instead of CEI has been introduced [4]. PIEB infusion is a method of bolus administration that injects a preset volume of the drug into the epidural space at regular intervals via an automated infusion pump. Theoretically, intermittent bolus administration increases the extent of neural blockade and decreases unilateral blockade, thereby improving the quality of epidural analgesia in postsurgical pain management [3].

In labor analgesia, recent studies have demonstrated a local anesthetic-sparing effect, lesser motor blockade, and higher maternal satisfaction with PIEB compared to that with CEI [5,6]. However, because of different pain entities and the target spinal level in major abdominal surgery compared with labor analgesia, the clinical application of PIEB in abdominal surgery might be limited. Few studies have compared the use of PIEB versus CEI after major abdominal surgery, [7,8] and two studies showed inconsistent postoperative outcomes. One study showing the difference between the two groups reported that the PCEA bolus needs were decreased in the PIEB group. Moreover, it is known that postoperative pain after upper abdominal surgery is the most severe among all major abdominal surgeries [9]; however, there is no study that has compared the two epidural methods in major upper abdominal surgery.

Therefore, we aimed to retrospectively compare the clinical effects and safety of PIEB versus CEI in combination with PCEA boluses for postoperative analgesia after major upper abdominal surgery. Our primary outcome was the cumulative rescue analgesic consumption at 72 h after surgery. In addition, we compared the 24 and 48 h postoperative rescue analgesic dose, epidural opioid and local anesthetic dose, pain score, and incidence of adverse events such as postoperative nausea and vomiting (PONV), hypotension, and pruritis between patients treated with PIEB and CEI.

## 2. Materials and Methods

### 2.1. Patients

The protocol of this retrospective study was approved by the institutional review board of Asan Medical Center (2020–1926). The study included patients who were scheduled for major upper abdominal surgery under planned thoracic epidural analgesia between July 2018 and October 2020. Patients included in the study were adults aged 18 years or above and scheduled for upper abdominal surgery due to hepatobiliary diseases. The patients with surgical incisions for upper abdominal surgeries from a xiphoid process (T6 dermatome) to umbilicus (T10 dermatome) were included. The incisions consisted of a vertical midline with/without a low transverse extension. Hepatectomy, bile duct resection, pylorus preserving pancreaticoduodenectomy, distal pancreatectomy, and Whipple’s procedure were included in the present study. Exclusion criteria included, unilateral blocks requiring catheter manipulation or replacement, catheter removed unintentionally, chronic pain or daily opioid consumption exceeding 100 morphine milligram equivalents (MME) for >14 consecutive days before surgery, open and closure procedure, and transfer to the intensive care unit.

### 2.2. Ultrasound-Assisted Thoracic Epidural Catheter Placement

After arriving in the operating room the day before surgery, routine monitoring was performed before the procedure. Pre-procedural ultrasound scan and marking of the insertion site were performed with the patient in a prone position with a pillow under the upper abdomen to increase the target interlaminar space. After preprocedural scanning of the interlaminar space between T9 and T12, the interlaminar space with the best visualization of epidural structures such as the ligament flavum, posterior dura, and anterior complex (anterior dura, posterior longitudinal ligament, and vertebral body) was considered the target space. Per our standard of care, the T10–T11 interspace is most commonly selected for ultrasound-assisted thoracic epidural catheter placement [10]. A high-frequency linear ultrasound probe (12 MHz; NextGen LOGIQe, GE Healthcare, Madison, WI, USA) was used. After obtaining a paramedian sagittal oblique view for the best visualization of the posterior complex (i.e., the ligamentum flavum and posterior dura), an 18-gauge Tuohy needle (Perifix, B. Braun Melsungen AG, Melsungen, Germany) was inserted from the caudal end of the probe and advanced in-plane view under real-time ultrasound-guidance until the needle tip reached in front of the posterior complex in the interlaminar space. When loss of resistance was felt, the epidural catheter was inserted. Subsequently, to rule out intravascular or intrathecal placement and check the level of the covered dermatome, 4 mL of 1% lidocaine was administered through the epidural catheter as a test dose. The extent of sensory blockade was assessed by cold sensibility testing to evaluate the appropriate spread of the epidural block.

### 2.3. Epidural PCA Setting

Patients were categorized into two groups based on the PCEA maintenance technique. In the PIEB group, the regimen was a PIEB of 3–4 mL every 60 min. In the CEI group, analgesia was maintained with a CEI of 3–4 mL for 60 min. Both pump settings were combined with a PCEA bolus of 1.5–2 mL (lockout time: 20 min). The basal infusion was set to 4 mL/h if the dermatomes spread within the range of surgical incision when the test dose was administered and was set to 3 mL/h in other cases according to the protocols of our institution. In addition, the doses of PCEA bolus (1.5 vs. 2 mL) were determined following the same protocols. The automated infusion pump (Accumate1200; Wooyoung Medical, Jincheon-Gun, Korea) was used for epidural infusion. Depending on the patient’s age, weight, and covered dermatome level, the PCEA settings were determined at the discretion of the supervising anesthesia provider within our institutional protocol. At our institution, the epidural analgesia solution consists of 0.15% ropivacaine and sufentanil 0.6–0.8 μg/mL.

### 2.4. Anesthetic Management Based on the Enhanced Recovery after Surgery (ERAS) Protocol

Oral complex carbohydrates were administered 2–3 h before the induction of anesthesia to reduce the catabolic state induced by overnight fasting. To decrease the risk of aspiration, ultrasound evaluation of the gastric volume was performed before the induction of anesthesia. Anesthesia was maintained using a target-controlled infusion (TCI) of propofol and remifentanil to maintain a bispectral index of 40 to 60. To minimize the surgical stimulus, an epidural bolus dose of 5 mL of 0.15% ropivacaine was administered at least 20 min before the surgical incision. Epidural PCEA was initiated within 1 h after the bolus infusion. According to the ERAS (Enhanced recovery after surgery) protocol, infusion of intravenous (IV) fluid was maintained at near-zero balance, and a vasoconstrictor such as norepinephrine was used when hypotension was detected [11,12]. All patients received a standard IV dual antiemetic prophylaxis (dexamethasone and ondansetron) with regard to patient-specific conditions [13] and 1 g IV acetaminophen as a non-opioid analgesic component of our standard multimodal analgesic regimen [14]. According to the ERAS protocol, the inspired fractional concentration of oxygen should be titrated to produce normal arterial oxygen levels to prevent hyperoxia-induced lung injury [15]. Thus, the inspired fractional concentration of oxygen was maintained at 0.3–0.4% with a target partial pressure of arterial oxygen level of 100 mmHg. Active warming using Hot line (SIMS Level 1, Inc., Rockland, MA, USA) and air warmer was performed to prevent intraoperative hypothermia.

### 2.5. Postoperative Analgesia

Patients were educated about using the PCEA bolus whenever the numeric rating scale (NRS) was 4 or higher. In the case of an inadequate analgesic response to the PCEA bolus (to reach an NRS < 4 within 10 min after bolus administration), IV rescue analgesia with IV fentanyl (1 μg/kg) in the PACU and IV pethidine 25–50 mg or IV tramadol (1 mg/kg) in the ward was allowed as a rescue option. According to the ERAS protocol, non-steroidal anti-inflammatory drugs are essential in multimodal analgesia for postoperative pain management. The fifty mg of dexketoprofen was administered intravenously twice a day until taking the PO analgesics in the ward. In patients with a history of asthma, hepatic or renal impairment, the administration was excluded as contraindications.

### 2.6. Outcome Assessment

Our primary outcome, cumulative rescue analgesic consumption, was the sum of intravenous opioid consumption up to 3 days after surgery. Intravenously administered opioids were converted into IV morphine equivalents. (i.e., IV morphine 10 mg = IV hydromorphone 1.5 mg = IV tramadol 100 mg = IV fentanyl 100 mcg = IV pethidine 75 mg) based on the previously published literature [16]. Total amount of local anesthetics and opioid administered via epidural including basal amount and PCEA boluses were checked at 24 h intervals during POD 1, 2, and 3. Patients were educated about NRS score evaluation the day before surgery and pain score was evaluated using the single 11-point NRS (0 = no pain, 10 = worst imaginable pain). And postoperative pain was assessed up to 3 days after surgery as average and worst pain, measured by certified nurses on the acute pain service in the post-anesthesia care unit (PACU) and ward, respectively. The secondary outcome, hypotension was based on MBP < 65 mmHg, and vasopressor use was based on the use of norepinephrine and phenylephrine due to hypotension [17]. Neurologic deficits referred to newly developed paresthesia or motor weakness.

### 2.7. Statistical Analysis

Continuous variables are expressed as mean ± standard deviation (SD) for the normally distributed data or as medians (including the 25–75th interquartile range) for the non-normally distributed variables. Categorical variables are reported as frequency (percentage). An analysis of continuous variables was performed using the Student’s *t*-test for mean differences or the Mann Whitney U test when nonparametric testing was necessary. Fisher’s exact test for categorical variables or a chi-squared test were used when appropriate for evaluating the differences between the groups. *p* values ≤ 0.05 were considered to indicate statistically significant differences. Statistical analysis was performed using R Statistical Software (version 4.0; R Foundation for Statistical Computing, Vienna, Austria).

### 2.8. Sample Size Calculation

To calculate the sample size of this study, we assumed the mean difference and standard deviations were 10 and 17.6 mg of opioid consumption in the 72 h after surgery between the groups, with a significance level of 0.05 and a power of 0.9, and then 69 patients per group were sufficient. To meet the sample size of PIEB group needed (69 patients), 124 patients in CEI group were included in this study.

## 3. Results

A total of 223 patients who received thoracic epidural analgesia during the study period were identified. Of these, 30 patients were excluded due to the following reasons: open and closure (*n* = 19), transfer to the intensive care unit (*n* = 3), chronic pain or daily opioid consumption exceeding 100 MME for >14 consecutive days before surgery (*n* = 3), catheter removed unintentionally (*n* = 2), asymmetric or unilateral block requiring catheter manipulation (*n* = 1), incomplete documentation (*n* = 1), conversion to IV PCA (*n* = 1). Thus, a total of 193 patients were included in the final analysis, of which 124 patients received CEI, and 69 patients received PIEB. Among the 193 patients, the baseline characteristics showed no significant differences between the groups, with the exception of ASA class (Table 1). ASA class Ⅰ was significantly higher in the CEI group whereas, ASA class Ⅲ was significantly higher in the PIEB group. However, the difference in outcome variables (total opioid consumption, pain score, epidural local anesthetics dosage) according to the difference in ASA classification was not significant (Table 2). All epidural catheters were inserted in the thoracic spine between T9-11 levels. The median duration of epidural therapy, ropivacaine concentration, and sufentanil dose was not significantly different between the groups. Intraoperative variables such as duration of surgery, fluid balance, and estimated blood loss were comparable between the two groups.

**Figure 1:**
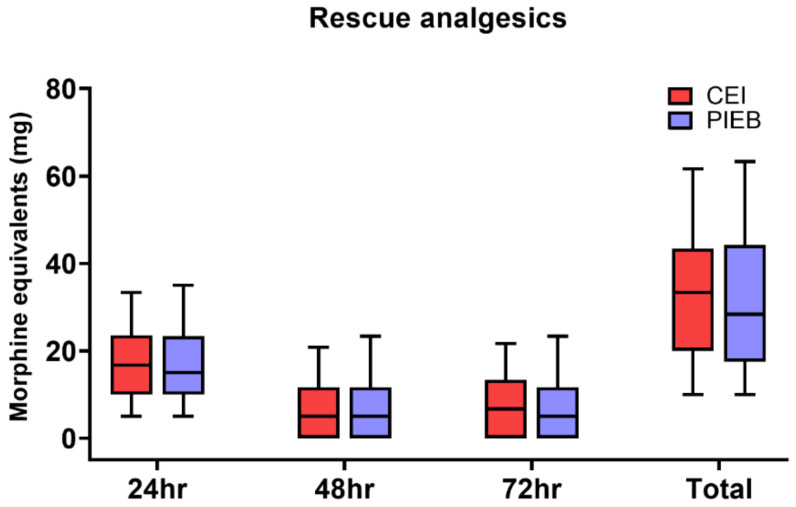
Postoperative rescue opioid consumption during the 72 h postoperative period and total cumulative consumption between the PIEB and CEI groups. CEI, continuous epidural infusion; PIEB, programmed intermittent epidural bolus. Results are expressed as box and whisker plots, including median, 10th, 25th, 75th, and 90th percentiles.

The need for rescue analgesic dose for the first 72 h was calculated separately. There was no significant difference in the rescue opioid consumption administered intravenously in the 72 h postoperative period between the groups (33.3 mg [20.0–43.3] vs. 28.3 mg [18.3–43.3], *p* = 0.375) (Figure 1). The dose of rescue analgesic was higher in the CEI group than in the PIEB group in the PACU (7.9 mg [3.3–10.0] vs. 5.0 mg [0.0–8.3], *p* = 0.015). However, there were no differences in the dose of rescue analgesic between the two groups in the first 24 h (CEI: 16.7 mg [10.0–22.9] vs. PIEB: 15.0 mg [10.0–23.3], *p* = 0.530), 48 h (5.0 mg [0.0–11.7] vs. 5.0 mg [0.0–11.7], *p* = 0.939) and 72 h (6.7 mg [0.0–13.3] vs. 5.0 mg [0.0–11.7], *p* = 0.439) after surgery (Figure 1). Other non-opioid analgesics doses were also not different until 72 h after surgery (*p* = 0.462).

**Figure 2:**
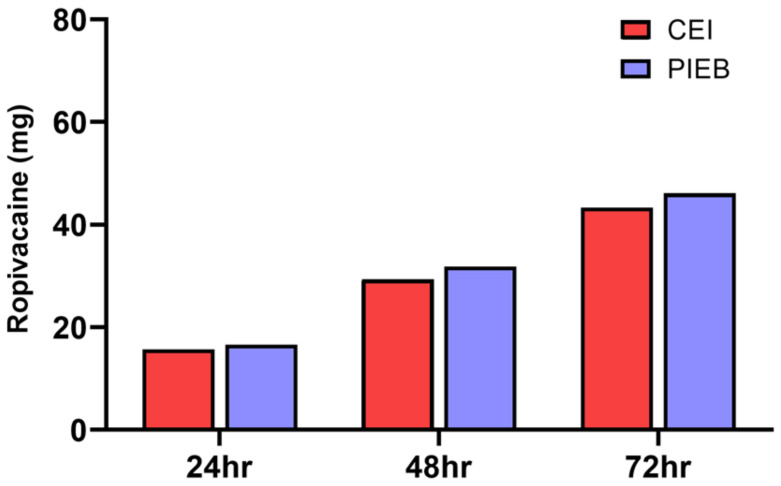
Cumulative consumption of the epidural local anesthetic during the 72 h postoperative period between PIEB and CEI. There was no difference in the epidural local anesthetic consumption between the two groups in 24 h, 48 h, and 72 h after surgery. CEI, continuous epidural infusion; PIEB, programmed intermittent epidural bolus. Data are visualized as bar graph with mean values.

There was no difference in the cumulative local anesthetic consumption between the two groups in 24 h (CEI 15.7 ± 4.6 mg vs. PIEB 16.6 ± 4.1 mg, *p* = 0.165), 48 h (29.4 ± 8.8 mg vs. 31.8 ± 7.9 mg, *p* = 0.060) and 72 h (43.4 ± 12.8 mg vs. 46.1 ± 11.4 mg, *p* = 0.130) after surgery (Figure 2).

**Figure 3:**
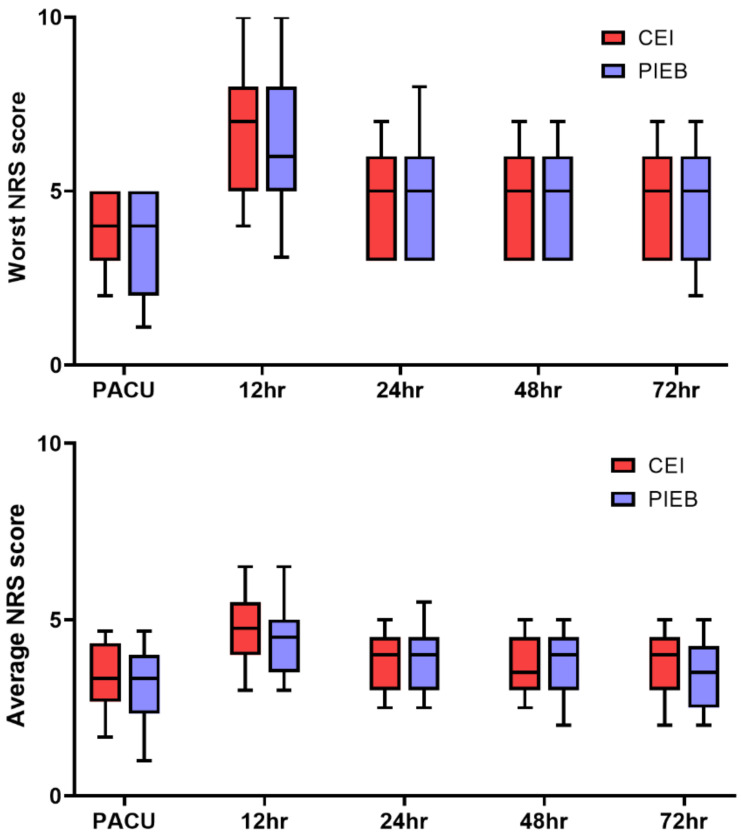
Comparison of pain intensity in the PACU at 12, 24, 48, 72 h after surgery between PIEB and CEI groups. A. Comparable worst pain scores between the PIEB and CEI groups. B. Comparable average pain scores between the PIEB and CEI groups. CEI, continuous epidural infusion; PIEB, programmed intermittent epidural bolus; NRS; numeral rating scale; PACU, post-anesthesia care unit. Results are expressed as box and whisker plots, including media, 10th, 25th, 75th, and 90th percentiles.

There was also no difference in the sufentanil consumption via epidural infusion between the two groups in 24 h (CEI: 63.8 μg [53.7–77.3] vs. PIEB: 56.1 μg [48.9–81.8], *p* = 0.289) and 48 h (92.8 μg [79.9–102.6] vs. 91.8 μg [55.9–106.1] *p* = 0.135) after surgery. The worst and average reported NRS value did not differ significantly between the groups for each given time point (Figure 3).

Patients in both groups encountered similar incidences of commonly reported adverse events associated with epidural analgesia (Table 3).

## 4. Discussion

To the best of our knowledge, this is the first study that has investigated the different modes of epidural application in only major upper abdominal surgery, and the number of enrolled patients was the highest, although it was a retrospective study. This study demonstrated two key outcomes. First, there was no difference in the quality of analgesia between PIEB and CEI after upper abdominal surgery. The study showed that postoperative rescue opioid consumption up to 72 h after surgery did not significantly differ between the groups. There was no difference in the overall pain scores between the groups for each given time point until 72 h after surgery. Additionally, local anesthetics consumption showed comparable values. Second, there was no difference in the incidence of complications between the two groups. These results suggest that both PIEB and CEI could safely provide postoperative analgesia after upper abdominal surgery.

Deciding the mode for effective and safe epidural analgesia might differ depending on the type and extent of surgery, the pattern of pain, and the level of insertion of the epidural catheter. In labor analgesia, previous studies reported that PIEB has the benefit of reducing the risk of breakthrough pain and improving maternal satisfaction while decreasing the amount of local anesthetics and rescue opioid analgesics required [18,19]. This might be because labor pain is intense in a short period of time, and the pain progressively descends from the lumbar to the sacral region as the delivery progresses. In thoracic surgery, PIEB reduced local anesthetic consumption but resulted in frequent hypotension [20]. Previous studies in abdominal surgery included various types of surgeries such as gynecology, upper and lower abdominal surgery; thus, the pattern of pain and level of insertion of the epidural catheter varied accordingly [7,8]. However, our study focused on major upper abdominal surgery only; consequently, our results are more reliable and reproducible in upper abdominal surgery compared to those of previous studies.

Rescue analgesic consumption between the two groups in PACU was statistically different, but the 24 h opioid consumption including the value in PACU did not differ between the two groups. Therefore, although it seemed to be statistically significant, the difference may not have clinical significance. The 24, 48, 72 h and total opioid consumption did not differ between the two groups. In addition, there were no differences in the doses of other non-opioid analgesics between the two groups at each time point. These results are consistent with previously published data showing that both PIEB and CEI epidural analgesia provided equally efficacious postoperative analgesia after major abdominal surgery [8]. Theoretically, because of the larger volume delivered with a bolus, PIEB techniques could provide superior analgesia through more extensive distribution of the epidural drug compared to that with continuous infusion [21]. Thus, PIEB techniques might have the advantage of extensive analgesia through a more efficient distribution of the drug, improving perioperative pain control, and reducing the requirement of analgesics [3,22]. However, in upper abdominal surgery, our results and previous studies did not show these advantages of PIEB.

There are several potential explanations for this discrepancy. First, more than 5 mL of bolus injection prior to CEI might result in a greater area of coverage by CEI [23]. Thus, the extent of neural blockade of CEI was comparable to that of PIEB in this study. Second, continuous infusion (basal rate ≥ 3 mL) could provide adequate analgesia over four dermatomes [24], relevant to the upper abdominal incision (T6–T9) [25]. Based on these findings, we conjecture that the analgesic effect of CEI might be similar to that of PIEB, at least in upper abdominal surgery. Third, the multimodal analgesic strategy per the ERAS protocol could minimize the superiority of PIEB over the CEI in postoperative pain management, with no difference in analgesic effect between the groups.

An optimal PIEB regimen with respect to the programmed bolus volume, time interval between doses, and concentration of local anesthetic, which vary significantly between the studies investigating PIEB has not been established [26,27]. Moreover, the optimal regimen can be changed depending on the surgical range, patients characteristic (age, sex, and height), and insertion level of the epidural catheter. An extensive midline abdominal incisions covered by several thoracic nerves (T5-12) must be blocked to provide sufficient analgesia. Therefore, it is necessary to define the optimal PIEB setting for various surgeries. Previous studies have shown that 0.2% ropivacaine provides the best balance of epidural analgesia with minimal motor block after major abdominal surgery [28]. Further, it is more effective to add opioids such as sufentanil to decrease the concentration of ropivacaine than to use 0.2% ropivacaine alone [29]. Thus, we used the combination of sufentanil 0.6–0.8 μg/mL and 0.15% ropivacaine in the study. Further studies are necessary to understand the optimal PIEB setting for upper abdominal surgery using different dosages of local anesthetics and opioids and varied programmed settings.

No significant differences were found in the incidence of adverse events in the two groups. The adverse effects of epidural analgesia appear to be related to the prolonged and extended sympathetic blockade [30]. In our study, the fluid input (crystalloid, colloid, and red blood cell) and output (urine output and estimated blood loss) during the intraoperative period were not different in the two groups. There was no difference in the rate of hypotension between the two groups. These results were consistent with those of a previous study [7], showing that the occurrence of hypotension and the need for vasopressor was not significantly different in both groups. The two groups did not differ in the incidence of PONV at any time point. This might be because total intravenous anesthesia and two or more antiemetics were used within the ERAS protocol. Our study was conducted within the recommendations of the ERAS protocol, a multimodal strategy aimed to improve the functional recovery after surgery [12]. The use of epidural analgesia is a vital part of the ERAS protocol in open abdominal or thoracic surgery because it blunts the neuroendocrine response to surgical stress, and allows better analgesia and faster ambulation [31]. In this study, transient sensory impairment of the lower extremities occurred in 2 out of 193 (1%) patients without any difficulties in ambulation. Therefore, both PIEB and CEI could be safe and effective analgesic methods that facilitate early mobilization after upper abdominal surgery under the ERAS protocol.

Our study has some limitations. First, because this was a retrospective analysis based on documentation in the electronic medical records, some values might be inaccurate or missing. Second, demographic data such as the ASA score showed a difference between the two groups. This was thought to have occurred because the sample size was not large enough. In the literature, pre-existing pain, anxiety, age, and type of surgery are generally regarded as significant factors affecting postoperative pain, but the association between ASA classification and postoperative pain is unclear [32,33]. Thus, we believe that this difference in ASA classification minimally affected the outcomes of the present study. Several confounding factors, including ASA score, were present and were not adjusted because of the retrospective designed study; they could limit the power of our study and the robustness of our conclusions. Therefore, the results of our study should be interpreted with caution, and a further, well-designed prospective randomized study is needed. Third, after epidural catheter insertion, the values of PCA setting were varied according to the dermatomal spread of the test dose. These lack of consistency of epidural PCA setting could be confounding factors although there was no statistical difference in the PCA setting. Finally, the aim of postoperative pain relief is to enhance the restoration of function by allowing the patient to breathe, cough, and ambulate easily [34]; however, pain assessment could not be conducted in detail because it was a retrospective study.

## 5. Conclusions

In conclusion, no differences were observed in the postoperative rescue opioid consumption, the dose of epidural local anesthetic, pain intensity, and adverse events between the PIEB and CEI groups. Therefore, we concluded that both PIEB and CEI approach in thoracic epidural analgesia could provide similar quality of analgesia and comparable safety after major upper abdominal surgery. However, the use of PIEB requires further evaluation according to the different settings of PIEB.

## Figures and Tables

**Table 1:** Patient characteristics and perioperative variables.

	CEI (*n* = 124)	PIEB (*n* = 69)	*p* Value
Age (year)	65.0 (58.0–71.0)	65.0 (61.0–69.0)	0.567
Gender, male	85 (68.5)	46 (66.7)	0.914
BMI (kg/m^2^)	23.6 ± 3.1	23.4 ± 3.2	0.692
ASA PS			0.007
I	13 (10.5)	2 (2.9)	
II	95 (76.6)	47 (68.1)	
III	16 (12.9)	20 (29)	
HTN	48 (38.7)	27 (39.1)	0.999
DM	33 (26.6)	25 (36.2)	0.218
IHD	9 (7.3)	9 (13.0)	0.286
CVA	7 (5.6)	7 (10.1)	0.387
Variables related to TEA			
Duration of epidural analgesia (h)	90.0 (87.7–94.2)	88.0 (81.0–93.0)	0.059
Needle depth (cm)	5.8 (5.3–6.0)	6.0 (5.5–6.1)	0.373
Sufentanil dose (μg)	150.0 (150.0–150.0)	150.0 (100.0–200.0)	0.131
Intraoperative variables			
Type of surgery			0.451
Whipple’s procedure	23 (18.5)	6 (8.7)	
PPPD	63 (50.8)	44 (63.8)	
Total pancreatectomy	6 (4.8)	4 (5.8)	
Distal pancreatectomy	8 (6.5)	3 (4.3)	
Hepatectomy	18 (14.5)	9 (13)	
BDR	6 (4.8)	3 (4.3)	
Duration of surgery (min)	250.5 (191.5–335.5)	238.0 (196.0–283.0)	0.300
Crystalloids (mL)	1600.0 (1300.0–2300.0)	1600.0 (1150.0–2000.0)	0.283
Colloids (mL)	100.0 (0.0–500.0)	100.0 (0.0–500.0)	0.816
Red blood cell (units)	0.0 (0.0–0.0)	0.0 (0.0–0.0)	0.855
Urine output (mL)	227.5 (137.5–345.0)	250.0 (135.0–400.0)	0.607
Estimated blood loss (mL)	109.0 (81–420.0)	100.0 (83–621.0)	0.956

Results are expressed as mean ± SD, median (IQR), *n* (%). CEI, continuous epidural infusion; PIEB, programmed intermittent epidural boluses; BMI, body mass index; ASA PS, American Society of Anesthesiologists physical status; HTN, hypertension; DM, diabetes mellitus; IHD, ischemic heart disease; CVA, cerebrovascular accident; TEA, thoracic epidural analgesia; PPPD, pylorus preserving pancreaticoduodenectomy; BDR, bile duct resection.

**Table 2:** Outcome variables according to ASA classification.

	ASA Ⅰ (*n* = 15)	ASA Ⅱ (*n* = 142)	ASA Ⅲ (*n* = 36)	*p* Value
PIEB, *n* (%)	13 (86.7)	95 (66.9)	16 (44.4)	0.007
Total opioid consumption (mg)	33.3 (19.2–45.8)	31.7 (18.3–45.0)	29.2 (22.5–42.5)	0.913
Mean NRS 72 h after surgery	3.5 (3.0–4.2)	3.5 (3.0–4.5)	4.0 (3.0–4.5)	0.726
Worst NRS 72 h after surgery	5.0 (3.0–6.0)	5.0 (3.0–6.0)	5.0 (3.0–6.0)	0.740
Epidural LA dosage (mg)	45.9 (37.5–52.3)	43.6 (37.5–53.7)	45.1 (37.5–52.9)	0.901

Results are expressed as median (IQR), *n* (%). ASA, American Society of Anesthesiologists; PIEB, programmed intermittent epidural boluses; NRS, rating numeral scale; LA, local anesthetics.

**Table 3:** Incidence of adverse events.

	CEI (*n* = 124)	PIEB (*n* = 69)	*p* Value
Hypotension			
PACU	22 (17.7)	15 (21.7)	0.627
24 h	16 (12.9)	15 (21.7)	0.999
48 h	4 (3.2%)	3 (4.3)	0.999
72 h	1 (0.8)	1 (1.4)	0.999
Use of vasopressor			
PACU	15 (12.1)	9 (13.0)	0.999
24 h	3 (2.4)	3 (4.3)	0.759
48 h	3 (2.4)	2 (2.9)	0.999
72 h	2 (1.6)	2 (2.9)	0.941
Nausea and vomiting			
PACU	2 (1.6)	4 (5.8)	0.241
24 h	16 (12.9)	15 (21.7)	0.162
48 h	16 (12.9)	10 (21.7)	0.928
72 h	18 (14.5)	11 (15.9)	0.956
Pruritus	2 (1.6)	1 (1.4)	0.999
Neurologic deficit	2 (1.6)	0 (0.0)	0.750

Results are expressed as *n* (%). CEI, continuous epidural infusion; PIEB, programmed intermittent epidural boluses; PACU, post-anesthesia care unit.

## Data Availability

Not applicable.
